# Workload as a predictor of radiographer preliminary image evaluation accuracy

**DOI:** 10.1002/jmrs.803

**Published:** 2024-07-02

**Authors:** Cameron Brown, Anna Burck, Michael J. Neep

**Affiliations:** ^1^ Department of Medical Imaging Logan Hospital Meadowbrook Queensland Australia; ^2^ Department of Medical Imaging Royal Brisbane and Women's Hospital Herston Queensland Australia; ^3^ School of Public Health and Social Work Queensland University of Technology Brisbane Queensland Australia; ^4^ Institute of Health and Biomedical Innovation Queensland University of Technology Brisbane Queensland Australia

**Keywords:** Diagnostic imaging, general radiography, medical imaging, quality improvement, radiographer

## Abstract

**Introduction:**

Despite a demonstrated high accuracy and reported successful implementations, radiographer preliminary image evaluation (PIE) has been slow and infrequent in its rollout across Australia. A key barrier reported to hamper radiographer PIE service implementation is lack of adequate time to review radiographs and provide an accurate interpretation. This study sought to conduct a correlational analysis between radiographer imaging workload and PIE service accuracy.

**Methods:**

A total of 45,373 exams and 1152 PIE comments evenly distributed each month from January 1, 2022, to December 31, 2022, were reviewed. PIE comments were assessed for consistency with the radiologist's report. The imaging workload (average exams completed per hour) was separated into three, eight‐hour ‘shifts’ based on time of imaging. Correlational analysis was performed using linear regression models and assessed for normality using the Shapiro‐Wilks test.

**Results:**

The study reported no significant linear association between increasing average workload and reduced service accuracy (*P* = 0.136). It was however noted that when the average workload increased beyond 7 exams/hour, average service accuracy for PIE was always below 85%.

**Conclusion:**

This study has demonstrated that, although perceived, there is no statistically significant correlation between x‐ray imaging workload and radiographer PIE service accuracy. Consideration of this correlation to be a significant barrier to participation in such a service was not reported at this site.

## Graphical abstract

Lack of adequate time to review imaging has been identified as a key barrier in the implementation of a radiographer preliminary image evaluation (PIE) service. Statistical analysis evidences no significant correlation between average hourly imaging workload and average hourly PIE service accuracy.

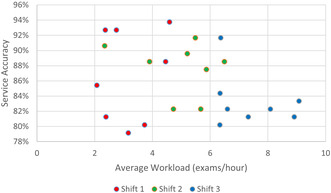

## Introduction

Within Australian emergency departments (ED), patient presentations continue to rise by approximately 6.9% each year since 2019.^1^ Since 2021–2022, only 61% of presentations were completed within the emergency length of stay (ELOS) window of 4 hours (a target of 80%),^2^ down from 67% in 2020–2021 and 71% in 2017–2018.^1^ During this time, medical imaging referrals within emergency departments have also steadily increased,^1^ increasing the delays in radiologist reporting turnaround times (RTAT).^3^

With links between RTATs and ED length of stay already established,^4^ prolonged interpretation of imaging has been proven to negatively affect emergency throughput, costs of care, length of stay and safety of care.^5^, ^6^ Numerous strategies have been implemented to minimise reporting delays in the emergency department. One such strategy proposed involves a radiographer documenting their interpretive opinion on the presence of potential pathology on the radiographs they acquire in addition to the authorised radiologist report. Principally, this interpretive opinion by the radiographer would be made available immediately to the referrer at the time of image acquisition which is particularly relevant when a radiologist report is delayed and outside an acute treatment window (such as an ELOS target). This system has been successfully implemented across the United Kingdom (UK) since 2004, reporting an accuracy of 92%.^7^ Australia has also adopted radiographer commenting at several sites (known as preliminary image evaluation, or PIE) reporting an identical accuracy (92%).^8^

Despite the reported benefits of the PIE system in Australia, national implementation has been infrequent and slow.^9^, ^10^ A 2018 national survey reported only 18% of respondents actively participate in a commenting scheme, despite 85% of the same respondents believing PIE contributes positively to patient safety.^11^ Significant educational, clinical and organisational support barriers have been identified in preventing increased participation in a PIE service by radiographers.^12^, ^13^ Namely, Neep et al.^12^ reports access to targeted image interpretation education (43%) and lack of time to review radiographs (41%) as the two most significant barriers that surveyed respondents believe inhibit the implementation of a successful PIE system.

Efforts to address targeted image interpretation have been positively received.^14^, ^15^, ^16^, ^17^ Literature evidences a strong correlation between radiographer PIE accuracy, perceived accuracy and confidence.^18^, ^19^ In particular, Williams et al.^14^ reported an accuracy improvement of 77.5% to 83.6% in appendicular skeletal abnormality detection in radiographers undertaking a short non‐intensive course. Neep et al.^15^ investigated the effect of intensive and non‐intensive image interpretation, reporting a 41% accuracy improvement across both groups. Furthermore, participants also reported increased confidence scores of 16% in abnormality detection, particularly after intensive education.

However, there is currently a paucity of evidence investigating the validity of the claim that radiographers ‘lack time to review’ their radiographs and provide a PIE comment. This indicates from respondents that workloads are too high to safely make the time to review and provide an accurate interpretation of the radiographer's findings. This may stem from various factors beyond workload alone, including the perception that PIE acts as an additional work burden, independent of comment accuracy. The calculated increased time burden of providing a PIE comment is an important, yet separate question to address. Regardless, it is not clear from respondents if the lack of time restricts the participating radiographer from providing an accurate analysis and comment or participating in the service at all, thus inhibiting service implementation. Both are separate issues each requiring due analysis and discussion of service accuracy and participation rates, respectively. The purpose of this study was to address the impact of this perceived barrier on PIE service accuracy. It was analysed by conducting a correlational study between the hourly imaging workload of an emergency department within Australia and the corresponding radiographer PIE accuracy.

## Methods

### Design

A retrospective study design was used to determine the correlational relationship between imaging workload and radiographer PIE accuracy between 1 January 2022 and 31 December 2022 at Logan Hospital, Queensland, Australia.

### Study setting

Logan Hospital is located in southeast Queensland. The hospital has 492 beds within the Metro South Hospital Health Service.^20^ From July 2021 to June 2022, 105,592 patients presented to the hospital's emergency department, the second‐largest intake in the state. Participants in the PIE portion of this study included all radiographers who worked in Logan Hospital's emergency medical imaging department within the study period (*n* = 48). The PIE service was operational 24 hours a day, 7 days a week throughout the study period.

### Audit sample size

**Figure 1:**
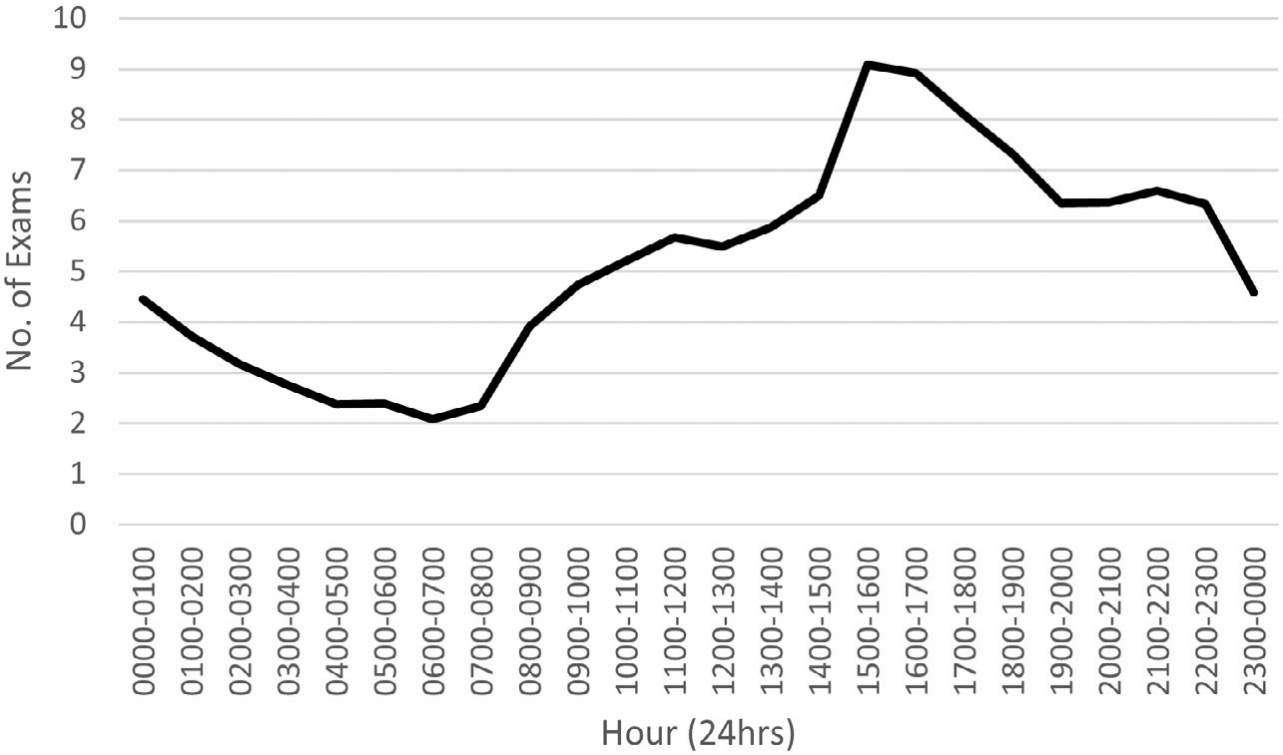
Linear histogram of hourly average workload over time.

Sample size calculation was conducted with an emphasis on ensuring a sufficient, randomised sample was obtained which was representative of the entire audit period and would allow an accurate correlation between the PIE accuracy and imaging workload datasets. To achieve this, it was decided to anonymise and obtain all x‐ray examinations within the audit period and separate the data by month of examination. Then, to ensure an accurate distribution of data across an entire working day, monthly x‐ray examinations were further categorised and grouped by hour of examination (e.g. 0600–0700, 1400–1500, 2300–0000). Imaging workload was collected as an average of the total x‐ray examinations performed within the audit period, grouped by hour (average hourly workload/hour). A total of 45,373 examinations were collected over the audit period (see Fig. 1). To acquire PIE accuracy data, four randomly selected examinations (within the scope of the site's PIE system) were acquired for each hour, across each month of the 12‐month period to analyse the PIE accuracy. This resulted in 96 PIE comments analysed from each month, evenly distributed over a 24‐hour period. A total of 1152 PIEs were collected over the audit period.

### Procedure

#### PIE accuracy analysis method

The procedure to analyse the PIE accuracy utilised the same methods employed by Brown et al.^8^ to assess PIE accuracy. Pathologies that were considered within the scope of the department's PIE system are listed in Table 1. An identical ‘ALERT’, ‘NO ALERT’ and ‘OUTSIDE SCOPE’ commenting framework to the Brown et al.^8^ study was also employed. This also includes a free text description only required for studies assigned by ‘ALERT’ to provide an imaging comment. ‘OUTSIDE SCOPE’ was only required for studies which queried pathologies outside the defined PIE scope (see Table 1).

**Table 1:** Scope of preliminary image evaluation pathologies.

The Radiographer's scope
A bony fracture
A joint dislocation or subluxation
A foreign body
A pneumothorax
A pneumoperitoneum
Knee lipohaemarthrosis or posterior elbow joint effusion

Any examinations which don't query any of these pathologies are outside scope and are not included in the audit.

PIEs were compared to the accompanying authorised radiologist report. Table 2 describes the confusion matrix developed by Brown et al.^8^; employing True Positive (TP), True Negative (TN), False Positive (FP), False Negative (FN), True Positive/False Negative (TP/FN) and Non‐Participation (NP) terms to classify PIE data with respect to the radiologist report. Categorisation, analysis and accuracy calculations of the PIE comment also aligns with the published work of Brown et al.^8^

**Table 2:** Confusion Matrix Terms List – Description of preliminary image evaluation classification following comparison to the radiologist report developed by Brown et al.^8^

True Positive (TP)
The radiographer's PIE and the radiologist's report agreed on the presence of an acute abnormality, and on the description of the abnormality in terms of injury, site and side. If multiple abnormalities were present, and one or more were missed by the radiographer, a TP score was not recorded. Similarly, credit was not given if the injury, site and side of the abnormality/ies was incorrectly described.
True Negative (TN)
The radiographer's PIE and the radiologist's report agreed on the absence of any acute abnormality. If a normal variant was identified by the radiographer and radiologist, a TN was allocated.
False Positive (FP)
The radiographer's PIE identified and described an appearance as abnormal; however, the radiologist's report disagreed and considered the examination to be normal, a FP was allocated.
False Negative (FN)
The radiographer's PIE identified an examination as normal; however, the radiologist's report stated that an acute abnormality was present.
True Positive/False Negative (TP/FN)
If the radiographer identified the correct abnormality but did not provide all key elements of the description (type of injury, site and side) or multiple abnormalities that were included in the radiologist's report; however, not all were identified, then fractions of marks (1/2) were awarded. For any case, where partial marks were awarded, the constituent partial marks amount to one mark. For example, if 1/2 TP was allocated, the remaining mark given was 1/2 FN.
Non‐Participation (NP)
If the imaging request was within the radiographer's scope and required a PIE, but no interpretation was provided; a No Participation value was assigned.

### Data analysis method

#### PIE service accuracy analysis

Resulting TP, TN, FP, FN, Unsure, Non‐participation and fractions were summed, and sensitivity, specificity and accuracy percentages were calculated by using the accepted confusion matrix formulae.^21^ Total service accuracy, the metric used to correlate with imaging workload, was calculated by subtracting the total accuracy percentage by the non‐participation and unsure percentages.

#### Workload correlation analysis

The hourly separation of data over a 24‐hour‐period metric is circular, not linear, with the 23^rd^ hour adjacent to the zero hour. This limits the authors' ability to normalise and validate the data. To compensate for this, the hourly data was instead separated into three, eight‐hour ‘shifts’. Shifts were separated and ordered as follows; 2300–0700 (1^st^), 0700–1500 (2^nd^), 1500–2300 (3^rd^). This aligns with the same rostered shifts at the study site. Boxplots displaying average, interquartile range (IQR), standard deviation, minimum and maximum values for service accuracy and workloads across each shift was then able to be generated.

Average workload and service accuracy were assessed for normality for each shift using the Shapiro‐Wilks test. Correlation between average workload and service accuracy were assessed with a linear regression model. The analyses were performed by the Molecular Biostatistics branch of the Queensland Cyber Infrastructure Foundation using the R software version R version 4.3.0 (2023‐04‐21) and developed in RStudio 2022.2.1.461.

### Ethics

Metro South Hospital and Health Service Human Research Ethics Committee provided ethical approval. This approval was included as part of a larger retrospective study under Originating Application ID: AU/1/540237. No personal identifying information was collected during this study.

## Results

**Figure 2:**
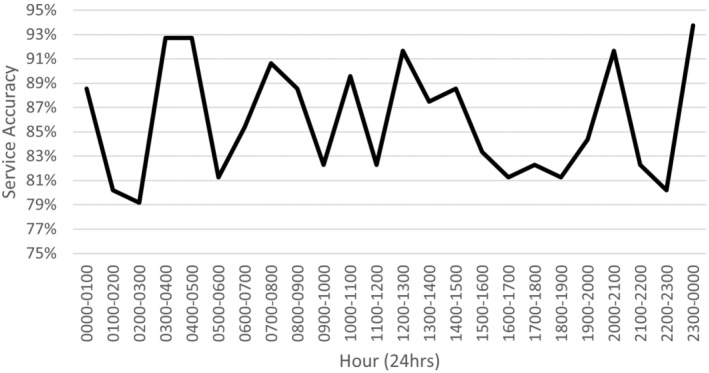
Linear histogram of hourly percentage service accuracy over time.

Over the 24‐hour period, hourly sensitivity ranged from 53.9 to 100%, hourly specificity ranged from 93.3 to 100%, hourly non‐participation rate ranged from 2.1 to 14.6%, hourly unsure rate ranged from 0 to 6.7% and hourly service accuracy ranged from 79.2 to 93.8%. See Figure 2 for a full illustration of hourly service accuracy over time.

**Figure 3:**
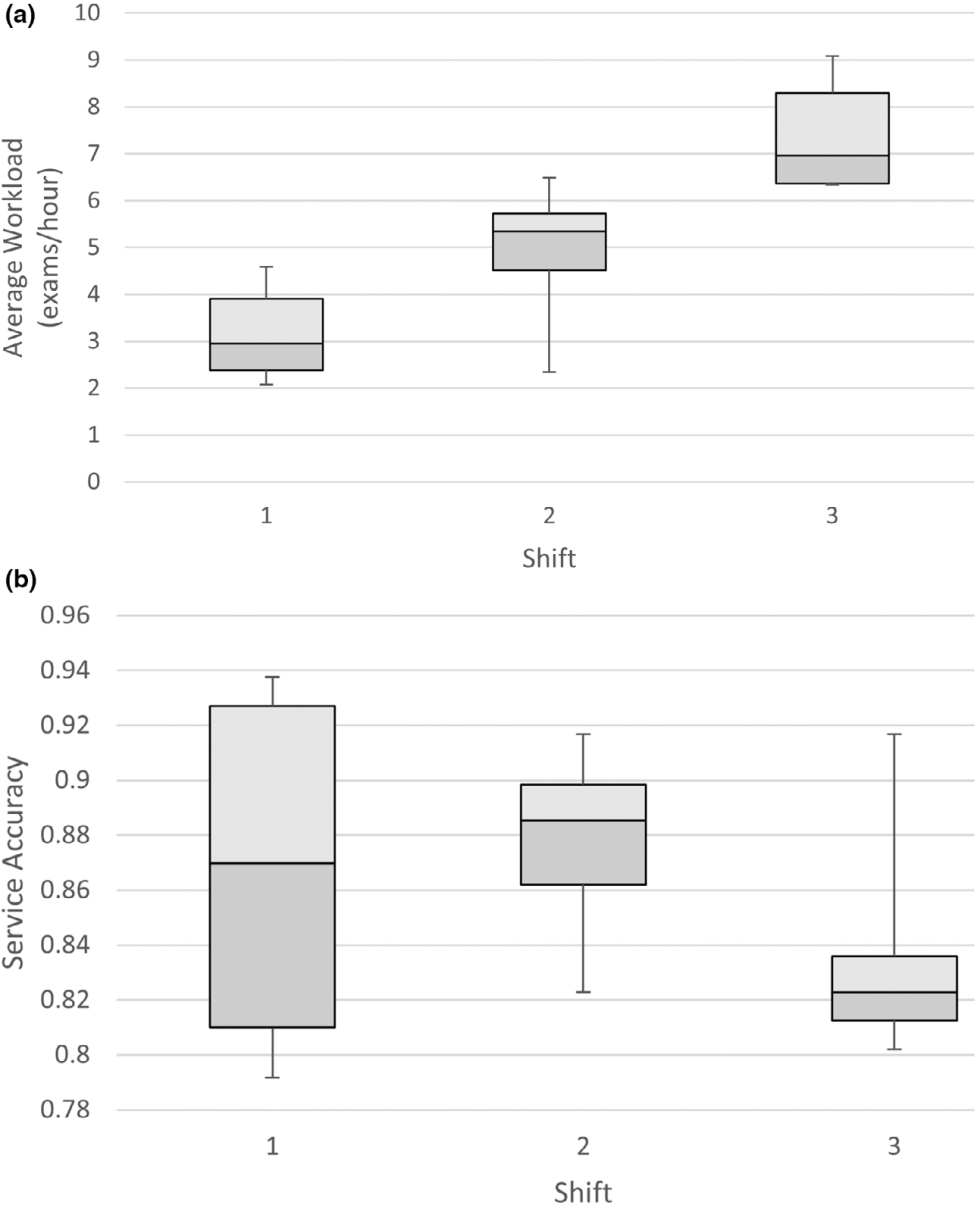
Boxplot of (a) average workload over shift and (b) service accuracy over shift.

There was a highly significant association between average workload and shift (*P* < 0.0001). The adjusted *R*‐squared was 0.689 indicating strong predictive power for individual shifts. The average workload increases with each shift and indeed almost increases by another 50% from shift to shift (see Fig. 3a).

There was no significant association between service accuracy and shift (*P* = 0.16). The adjusted *R*‐squared was 0.079 indicating weak predictive power for individual shifts (see Fig. 3b). Service accuracy was most variable during the first shift and then became more stable and differentiated for the second two shifts (Table 3).

**Table 3:** Tabulation of preliminary image evaluation accuracy data.

Time	0000–0100	0100–0200	0200–0300	0300–0400	0400–0500	0500–0600	0600–0700	0700–0800	0800–0900	0900–1000	1000–1100	1100–1200	1200–1300	1300–1400	1400–1500	1500–1600	1600–1700	1700–1800	1800–1900	1900–2000	2000–2100	2100–2200	2200–2300	2300–0000
TN	38	33	30	35	39	33	35	33	36	33	33	29	34	32	27	30	31	28	32	28	34	28	25	36
TP	4.5	5.5	8	9.5	5.5	6	6	10.5	6.5	6.5	10	10.5	10	10	15.5	10	8	11.5	7	12.5	10	11.5	13.5	9
FP	0	2	1	0	0	2	1	0	0	2	0	1	1	0	0	1	1	0	1	2	1	1	1	0
FN	0.5	3.5	6	2.5	1.5	0	2	1.5	4.5	2.5	4	2.5	2	5	3.5	4	4	2.5	6	2.5	2	6.5	2.5	2
Unsure	0	0	1	0	2	0	0	3	1	0	0	1	1	1	2	1	1	1	1	1	0	0	1	0
Non‐Participation	5	4	2	1	0	7	4	0	0	4	1	4	0	0	0	2	3	5	1	2	1	1	5	1
Sensitivity	90.00%	61.11%	57.14%	79.17%	78.57%	100.00%	75.00%	87.50%	59.09%	72.22%	71.43%	80.77%	83.33%	66.67%	81.58%	71.43%	66.67%	82.14%	53.85%	83.33%	83.33%	63.89%	84.38%	81.82%
Specificity	100.00%	94.29%	96.77%	100.00%	100.00%	94.29%	97.22%	100.00%	100.00%	94.29%	100.00%	96.67%	97.14%	100.00%	100.00%	96.77%	96.88%	100.00%	96.97%	93.33%	97.14%	96.55%	96.15%	100.00%
Non‐Participation	10.42%	8.33%	6.25%	2.08%	4.17%	14.58%	8.33%	6.25%	2.08%	8.33%	2.08%	10.42%	2.08%	2.08%	4.17%	6.25%	8.33%	12.50%	4.17%	6.25%	2.08%	2.08%	12.50%	2.08%
Service Accuracy	89%	80%	79%	93%	93%	81%	85%	91%	89%	82%	90%	82%	92%	88%	89%	83%	81%	82%	81%	84%	92%	82%	80%	94%

FN, False Negative; FP, False Positive; Non‐Participation, Failure to provide a PIE comment by participating radiographer; TN, True Negative; TP, True Positive; Unsure, Labelled ‘Unsure’ by participating radiographer.

**Figure 4:**
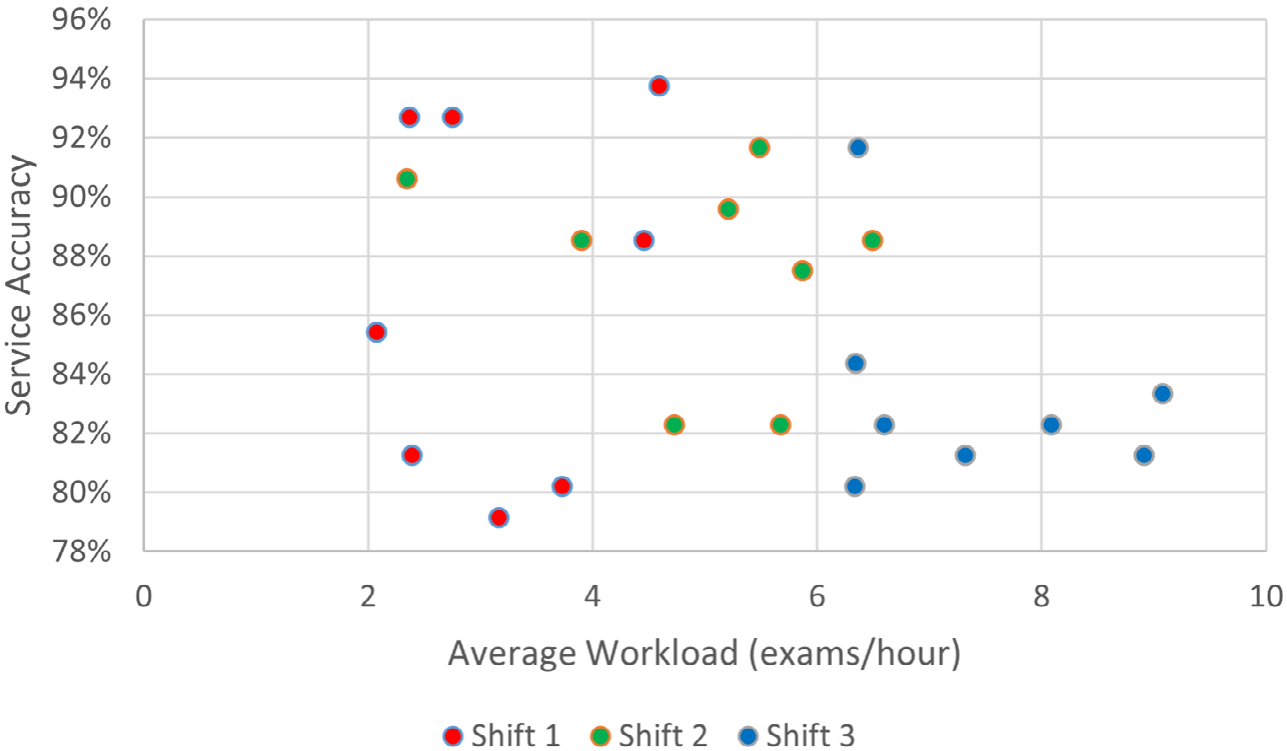
Scatterplot of service accuracy by average workload.

No linear trend was detected between increasing average workload and reduced service accuracy (*P* = 0.136). The adjusted *R*‐squared was 0.057 indicating poor predictive power for individual periods. There was too much variability for there to be a strong relationship. To be noted, there was a visual trend for decreasing service accuracy with increasing average workload but also apparent variability (Fig. 4). When the average workload increased beyond 7 exams/hour, average service accuracy was always below 85%. Furthermore, the majority of these occurred in the third shift.

## Discussion

This study addressed its intended aim to investigate the significance (if any) of the correlational relationship between radiographer imaging workload and PIE service accuracy. Linear regression modelling reported a statistically insignificant relationship (*P* = 0.136) between average workload and reduced service accuracy. It should be noted however, that a visual negative trend was visible in Figure 4, albeit variable. Also noteworthy, was a clear trough in service accuracy in Figure 2 aligning with a clear spike in average workload in Figure 1 at approximately 1500 hours. This coincides with comments by the biostatistical report which noted when average workload increased beyond 7 examinations per hour, service accuracy was always below 85%. Given service accuracy is the product of PIE accuracy, unsure interpretations and non‐participation combined, it is unclear if this trough in accuracy at high workloads is due to increased false negative and false positive PIEs or a reduced participation in the service. As previously mentioned, analysis into the effects of service participation rates with respect to workload is warranted in further research. Regardless, the biostatistical report concludes that no statistical relationship exists between average workload and service accuracy, either treating outcomes as continuous or categorical.

There also exists a lack of significant association between service accuracy and shift (*P* = 0.16). This indicates that the time of day the shift occurred had no effect on service accuracy. Interestingly, the variability of PIE accuracy was highest during the 1^st^ shift (2300–0700), which stabilised for the 2^nd^ (0700–1500) and 3^rd^ (1500–2300) shifts. This is despite the fact that staffing levels for the 1^st^ shift typically allocate one radiographer, with two allocated for the 2^nd^ shift and three for the 3^rd^ shift. Variability of service accuracy appeared to decrease with an increasing number of staff simultaneously participating in the service.

Given the paucity of statistical correlational data in assessing the validity of key PIE barriers, this study provides insightful exposition. It largely contradicts survey responses from radiographers who report the significance of this relationship being a key barrier to participation in and implementation of a PIE service.^12^ Throughout the 24 hour period, PIE service accuracy at Logan Hospital ranged from 79.17 to 93.75%, with a median accuracy of 84.90%. In comparison, a 2019 publication of PIE service accuracy at the same site was reported at 84.10%.^8^ This consistency illustrates a reproducible and highly accurate service that can be maintained under varying workloads.

Neep et al.^15^ addressed the barrier of lack of imaging interpretation education, by providing intensive and non‐intensive interpretation courses. This increased perceived radiographer confidence scores in PIE accuracy by 16%. Considering this, there is an opportunity in future research to assess and compare the perceived effect of workload on PIE by staff who directly participate in such a service.

There are several notable strengths and limitations of the study. Acquiring all x‐ray imaging examinations over the audit period (totalling 45,373 examinations) separated by hour of examination is a notable strength, this is contrasted by the lack of PIE service accuracy data. With only 4 examinations per hour, per month (totalling 1152 examinations), this represents only 2.5% of all examinations performed. Further analyses to a larger cohort of data would be prudent to validate this sample size. The decision to alter data analyses from hourly to three 8‐hour shifts provided clarity in data normalisation, especially given the cyclical nature of the data and the small PIE sample size. However, this lowered the specificity of the data, broadly allowing only shifts to be analysed for correlation and not specific hours. The authors were unable to accurately identify when key hours of the day are at risk of low PIE accuracy with statistical significance. Logan Hospital allocates one staff member for the 1^st^ shift, two for the 2^nd^ shift and three staff for the 3^rd^. This accurately aligns with increasing workloads, indicating the service has responded effectively to imaging demands. However, the presence of multiple and changing staffing levels is not considered when analysing the workload demand and PIE service accuracy. Given service accuracy variability decreases with increasing numbers of staff simultaneously contributing to it, further investigation into the cause of this is warranted.

Interesting areas of further research include the already mentioned mixed methods survey of key barriers from radiographers already participating in a PIE service to juxtapose the Neep et al.^12^ study. Furthermore, a detailed statistical investigation into the findings of <85% service accuracy at >7 exams/hour is warranted. One could assess if this is due to a reduction in PIE accuracy (increased FPs and FNs) or a reduction in service participation altogether (increased Unsures and NPs). This would guide feedback to the site and either target support for staff to increase accuracy or streamline avenues promoting more consistent involvement in the service.

## Conclusion

This study has demonstrated that, although perceived, there is no statistically significant correlation between x‐ray imaging workload and radiographer PIE service accuracy. There was, however, a threshold where service accuracy was always below 85% when workload increased beyond seven examinations per hour. With respect to PIE service accuracy and imaging workload demands alone, an existing PIE service can be maintained at an established accuracy standard. Given the success of the service at Logan Hospital in providing enhancement of the radiographer's role and improving safety within the multidisciplinary team, it is recommended to continue rolling this service out to sites across the country.

## Conflict of Interest

The authors declare no conflict of interest.

## Data Availability

The data that support the findings of this study are available on request from the corresponding author. The data are not publicly available due to privacy or ethical restrictions.
